# Enhanced prediction of expression control in bacterial biosynthetic gene clusters via genomic and functional data integration

**DOI:** 10.1099/mgen.0.001512

**Published:** 2025-10-15

**Authors:** Silvia Ribeiro Monteiro, Sébastien Rigali

**Affiliations:** 1 InBioS – Center for Protein Engineering, University of Liège, Institut de Chimie, Liège B-4000, Belgium

**Keywords:** biosynthetic gene cluster, natural product discovery, position weight matrix, regulatory network, transcription factor binding site

## Abstract

Accurate identification of transcription factor binding sites (TFBSs) is fundamental to understanding gene regulation. Traditional position weight matrix (PWM) motif-based methods scan genomes to predict potential TFBSs based on sequence similarity. However, these approaches struggle to detect degenerate or low-affinity sites, which are common in bacterial biosynthetic gene clusters (BGCs). BGCs, which encode proteins and enzymes for production, export, resistance and regulation of specialized bioactive compounds, are typically regulated by multiple transcription factors acting on weakly conserved binding sites. This complexity and regulatory specificity limit the effectiveness of standard motif-scanning tools, impeding efforts to activate silent cryptic clusters and discover novel natural products. To overcome these limitations, we developed COnditions for Microbial Metabolite Activated Transcription (COMMBAT), a scoring method designed to improve TFBS prediction in BGCs. COMMBAT integrates two complementary components: an *interaction score*, derived from PWM-based motif matching, and a *target score*, which incorporates both the genomic context (*region score*) and gene function (*function score*) within the transcriptional unit associated with the TFBS. These components are normalized and combined to generate a final *COMMBAT score* that more accurately reflects biological relevance, prioritizing TFBSs neighbouring promoter regions and regulating functionally important BGC genes (such as regulatory and core biosynthetic genes). Evaluations demonstrate that COMMBAT substantially outperforms sequence-only methods in identifying already experimentally validated TFBS, offering a powerful tool to accelerate the discovery of transcriptional elicitors of microbial natural product biosynthesis. The COMMBAT website is available at https://www.commbat.uliege.be

## Graphical abstract

COMMBAT integrates motif-based interaction and context/function-based target scores, generating a biologically relevant composite score for transcription factor binding sites.

## Data Summary

The authors confirm all supporting data, code and protocols have been provided within the article or through supplementary data files available with the online version of this article.

**Impact Statement**

In bacteria, identifying regulatory mechanisms that connect environmental signals to the expression of biosynthetic gene clusters (BGCs) is often hindered by the difficulty of accurately detecting transcription factor binding sites (TFBSs) within BGCs, where regulatory sequences are typically weak and poorly conserved. COnditions for Microbial Metabolite Activated Transcription (COMMBAT) addresses this critical bottleneck by integrating sequence-based motif detection with genomic and functional context to improve TFBS prediction accuracy. By enabling more reliable identification of functional regulatory elements in BGCs, COMMBAT (available at https://commbat.uliege.be) will facilitate the prediction of signalling pathways from environmental elicitors to BGC expression, offering a valuable prediction methodology for natural product discovery associated with cryptic BGCs.

## Introduction

The identification of transcription factor binding sites (TFBSs) is critical for elucidating the regulatory logic governing gene expression. Systematic mapping of these binding sites provides essential insights into the regulatory mechanisms that control biological processes and is particularly helpful in reconstructing transcriptional regulatory networks. These networks define how organisms coordinate the co-regulation of genes under fluctuating conditions, such as nutrient limitation, stress or host interaction. In bacteria, transcription factors (TFs) typically recognize and bind to short, specific DNA motifs in promoter-proximal regions to modulate transcriptional activity, making motif-based approaches common strategies for TFBS prediction [1]. DNA binding motifs are frequently represented using position weight matrices (PWMs), which quantify the likelihood of each nucleotide occurring at each position within a motif [2]. Genomic sequences are scanned using these matrices, and sites scoring above a defined threshold are designated as putative binding regions [3].

Accurate TFBS prediction is particularly relevant in the context of bacterial biosynthetic gene clusters (BGCs), as only an estimated 3% of the natural products associated with BGCs have been experimentally characterized [4]. TFBS mapping within BGCs can uncover key regulatory elements, providing insight into the activation of transcriptionally silent clusters for industrial and therapeutic purposes [5,6]. Importantly, BGC-associated TFBSs often show more divergent sequences compared to TFBS regulating genes within the TF’s core regulon and residing outside BGCs [7]. This reduced conservation reflects the need for BGCs to respond to diverse environmental cues, each mediated by a distinct TF. In such cases, lower-affinity TF binding may confer greater regulatory flexibility, allowing response to multiple environmental signals. However, this high level of TFBS degeneration poses a challenge for high-throughput screening, as these sites often fall below PWM thresholds [3]. Enhancing the accuracy of TFBS detection would aid in prioritizing BGCs for experimental validation, ultimately accelerating the discovery of novel bioactive compounds through targeted cultivation and genetic engineering.

In this study, we aimed to enhance the accuracy of motif-based TFBS prediction within bacterial BGCs by incorporating additional genomic and functional context into the scoring equation. Specifically, we developed the COnditions for Microbial Metabolite Activated Transcription (COMMBAT) scoring methodology, which integrates the predicted binding affinity of the TF and a contextual score reflecting both the genomic location and functional annotation of potential target genes. Evaluations of the COMMBAT scoring demonstrated a substantial improvement in the prediction of experimentally validated TFBSs compared to a method exclusively based on sequence similarity, highlighting the utility of biologically informed scoring strategies in regulatory site prediction.

## METHODS

### Data curation and analysis

To evaluate the developed COMMBAT methodology, known TFBSs within BGCs of ten TFs (AdpA, AfsQ1, BxlR, CebR, Crp, DasR, GlnR, MtrA, PhoP and SoxR) were collected from the literature. All the curated data, along with the referenced sources, are provided in Table 1. The sequence consensus logo was created for each TF with WebLogo3 (v2.8.2) [8]. The other visualizations were generated in R (v. 4.5.0) [9] using R packages ‘ggplot2’ (v. 3.5.2) [10] and ‘patchwork’ (v.1.3.0) [11]. The values of the different quartiles from the boxplot distributions were obtained with the function ‘boxplot.stats’ from the R package ‘grDevices’ (v.4.5.1) [9]. Shannon’s entropy information content (IC) was calculated as described in Schneider e*t al*. [12] using the R package ‘universalmotif’ (v. 1.26.2) [13].

**Table 1.** Comparison of the COMMBAT and interaction scoring methods of functional TFBSs

MIBiG BGC (compound)		TFBS sequence	Scores					Ranking progression	Ref
			*I*	*T* (*R*+*F*)	*R*	*F*	*C* (*I*+*T*)		
**AdpA**								seq nb: 3,803,784	
1	BGC0000020.3 (maytansine, ansamitocin P-3)	GTTCGGGCCA	0.64	0.55	1	0.1	0.59	44,262 -> 78,647	[22]
2	BGC0000253.4 (oviedomycin)	TGGCGCGACG	0.37	0.55	1	0.1	0.46	438,210 -> 473,511	[23]
3a	BGC0000325.5 (coelichelin)	GGGCCGATTC	0.59	0.9	1	0.8	0.74	74,052 -> 6,165	[24]
3b		GGGCCGATTC		0.75	1	0.5	0.67	74,314 -> 23,214	
4	BGC0000724.5 (streptomycin)	GAATCAGCCG	0.28	0.55	1	0.1	0.42	785,384 -> 739,519	[21]
5		TGGCGCGATC	0.78	0.55	1	0.1	0.67	5,958 -> 22,161	
6		TTTTCGGTCA	0.64	0.55	1	0.1	0.59	44,729 -> 79,401	
7		TGGCCGTTGC	0.33	0.55	1	0.1	0.44	552,654 -> 583,844	
**AfsQ1**								seq nb: 667,473	
8	BGC0001063.5 (prodiginines)	GATACGAAGTGGTTTC	0.38	1	1	1	0.69	17,780 -> 734	[25]
9	BGC0000315.5 (CDA)	CTGACCCGGCCGTAAC	0.38	1	1	1	0.69	17,776 -> 729	
10	BGC0000038.5 (coelimycin)	GTCACGGCGTGGTAAC	0.6	0.9	1	0.8	0.75	1,118 -> 172	[26]
**BxlR**								seq nb: 944,325	
11	BGC0000834.5 (cathomycin)	CGAATCATTCG	0.7	0.9	1	0.8	0.8	286 -> 87	[27]
**CebR**								seq nb: 195,118	
12a	BGC0002089.2 (thaxtomin)	CGGGAGCGCTCCCA	0.9	0.65	0.3	1	0.78	12 -> 21	[19]
12b				0.55	0.3	0.8	0.73	13 -> 48	
13		GGGGAGCGCTCCCA	0.9	0.55	0.3	0.8	0.73	14 -> 49	
**Crp**								seq nb: 2,150,629	
14	BGC0000315.5 (CDA)	GTACGCGATGTCAC	0.31	0.55	1	0.1	0.43	153,368 -> 198,299	[28]
15		GGGGTGCCGTACAC	0.26	0.55	0.3	0.8	0.41	252,091 -> 270,334	
16	BGC0000194.5 (actinorhodin)	GTGACGAGCGACGA	0.1	0.65	0.3	1	0.38	984,370 -> 448,227	
17a	BGC0000038.5 (coelimycin)	AAGATTCTCCTCAC	0.06	1	1	1	0.53	1,336,427 -> 50,838	
17b		AAGATTCTCCTCAC		0.75	1	0.5	0.41	1,341,449 -> 300,263	
18		GTGTCCGGCGGCGC	0.21	0.75	1	0.5	0.49	300,026 -> 88,904	
19	BGC0001063.5 (prodiginines)	GTGGTGGGCGAGAC	0.48	0.65	0.3	1	0.56	23,203 -> 24,571	
20	BGC0000660.5 (albaflavenone)	GTCGCGATACTCAC	0.18	0.4	0.3	0.5	0.29	54,5537 -> 1,205,282	
**DasR**								seq nb: 94,453	
21	BGC0001063.5 (prodiginines)	AGTGGTTTCCACCTCA	0.44	1	1	1	0.72	435 -> 22	[29,30]
22		ACTGCTGGAGACCGGT	0.16	0.65	0.3	1	0.41	16,650 -> 10,503	
23	BGC0000194.5 (actinorhodin)	TGTTGAGTAGGCCTGT	0.34	1	1	1	0.67	1,909 -> 84	
24	BGC0000038.5 (coelimycin)	ACATGCGTAATCAACT	0.22	0.9	1	0.8	0.56	8,665 -> 767	
25	BGC0000336.5 (daptomycin)	AGTGGTTTGGTCCGCC	0.29	0.9	1	0.8	0.59	3,706 -> 416	[31]
**GlnR**								seq nb: 669,027	
26	BGC0000136.5 (rifamycin)	TGAACACTTGTTTGAC	0.19	1	1	1	0.59	86,747 -> 4,153	[32]
27	BGC0000194.5 (actinorhodin)	TTAATTTTTGATCAAT	0.24	1	1	1	0.62	47,384 -> 2,355	[33]
28	BGC0001063.5 (prodiginines)	GATACGAAGTGGTTTC	0.24	1	1	1	0.62	47,315 -> 2,243	
**MtrA**								seq nb: 1,476,806	
29	BGC0000194.5 (actinorhodin)	GTCGCCCCCAGGAGAC	0.09	1	1	1	0.55	698,288 -> 25,632	[34,35]
30a	BGC0000038.5 (coelimycin)	GTCACGGCGTGGTAAC	0.7	0.9	1	0.8	0.8	335 -> 72	
30b				0.75	1	0.5	0.72	339 -> 617	
31	BGC0000315.5 (CDA)	CTGACCCGGCCGTAAC	0.58	1	1	1	0.79	2,457 -> 110	
**PhoP**								seq nb: 242,762	
32	BGC0000315.5 (CDA)	CGAGAGCGACC-GGCAGGTAACC	0.3	0.65	0.3	1	0.47	4,015 -> 9,153	[36]
**SoxR**								seq nb: 185,224	
33	BGC0000117.5 (oligomycin)	GCTCACTGATGTTTGACT	0.21	1	1	1	0.6	6,496 -> 393	[37]

*I*, interaction score; *T*, target score; *R*, region score; *F*, function score; *C*, COMMBAT score. Colour code: blue to red scale indicates values negatively and positively impacting the COMMBAT score, respectively. seq nb represents the number of predicted sites matching the TFBS length identified across BGCs in the MIBiG database through motif scanning with a PWM similarity score >0. The ranking progression shows the rankings of TFBSs with the interaction and COMMBAT scorings, respectively. The cited references report the identification of the mentioned TFBSs within the respective BGCs.

### PWM creation and TFBS identification and scoring

PWMs were generated for ten TFs using known TFBSs from their respective target genes that reside outside BGCs, which are available in File S1, available in the online Supplementary Material. The PWMs were built according to the expression described by Hertz and Stormo [2]. For each TFBS, the PWM score was calculated with the PREDetector software [14] and was converted into an interaction score (*I*). The latter is normalized between 0 and 1 (with 1 representing the consensus sequence) using the expression described by Ribeiro Monteiro *et al*. [7]: *I*_TFBS_/*I*_max_

where *I*_TFBS_ is the PWM score given for a specific TFBS, and *I*_max_ is the maximum score of the PWM (consensus sequence).

### Prediction and ranking of TFBSs

For each TF, we scanned all the 802 *Streptomyces* BGCs from the MIBiG database (v. 4.0, May 2025) [15], together with the non-*Streptomyces* BGCs regulated by these TFs, with either the COMMBAT (this work) or PREDetector [14] method. From this set of BGCs and for each TF, we collected all the sequences that matched in length to their TFBSs, with a similarity PWM score >0. Using a low cutoff score of 0 ensured inclusion of a broad pool of false-positive binding sites for each TF. The ranking of the experimentally validated TFBSs within this pool was determined for each methodology and reported in Table 1. The rankings were then normalized by dividing the ranking of the TFBSs by the total number of predicted sites (and multiplied by 100 to express the values as percentages). To assess the ranking progression of each functional TFBS, we calculated the ranking difference of that TFBS between the COMMBAT and interaction scoring methods and normalized this ranking difference by the total number of predicted sites (again expressed as percentages).

## Results and discussion

### Limited effectiveness of motif-based TFBS prediction in bacterial BGCs

**Fig. 1.**
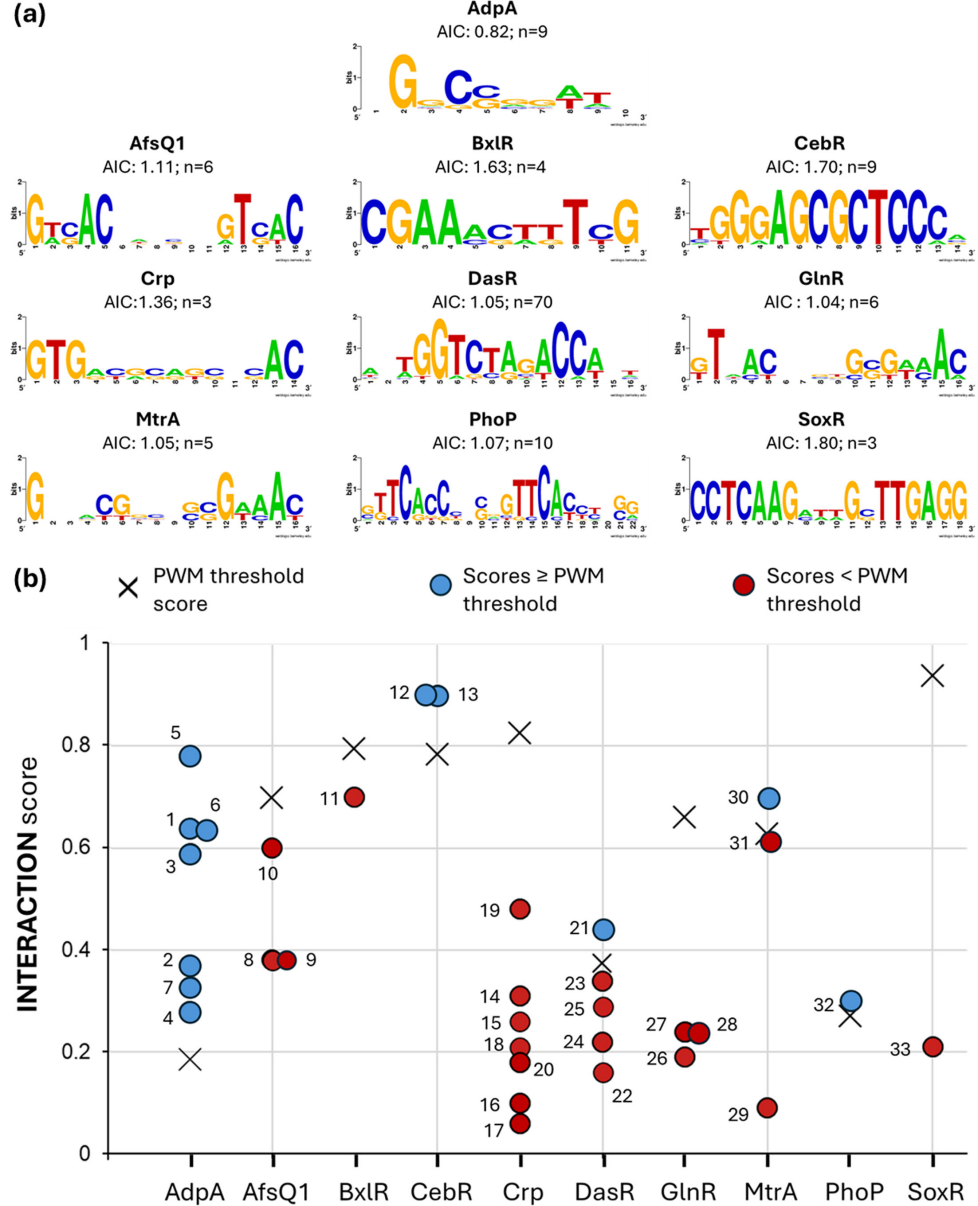
Interaction score of TFBS in bacterial BGCs. (**a**) Average information content (AIC) for TFBSs of the ten TFs selected for this study (maximum AIC value is 2). The conservation and frequency of each nucleotide at each position of the TFBS are illustrated by a sequence logo generated with WebLogo3 [8]. (**b**) Comparison of the interaction score of TFBS in BGCs from Table 1. The interaction score reflects the strength of the binding affinity of a TF for a TFBS. Scores are calculated as the ratio of the PWM score of a predicted TFBS to the maximum PWM score for each TF. Scores are normalized on a scale from 0 to 1, where a score of 1 corresponds to the consensus sequence, theoretically representing the highest binding affinity for the TF. The ‘X' symbol represents the PWM threshold, and the green and red dots are TFBSs with a score above and below the PWM thresholds, respectively.

The production of specialized metabolites encoded by bacterial BGCs is typically regulated in response to diverse environmental signals, each mediated by a specific TF [16,18]. To integrate multiple regulatory inputs and avoid strict control by a single TF, TFBSs within BGCs often deviate more from the optimal consensus sequences recognized by their corresponding TFs than those found in genes of the TF’s core regulon and that reside outside BGCs [7]. As a result, these suboptimal binding sites are more difficult to detect using standard motif-based prediction methods. To assess this limitation, we generated the PWMs for ten TFs using known TFBSs associated with genes that reside outside BGCs and evaluated their ability to detect experimentally validated TFBSs within BGCs. The selected TFs exhibit varying tolerance (IC) to sequence deviations from their consensus binding motifs (Fig. 1a). Some, such as CebR, are highly sequence-specific, whereas others, such as AdpA, can bind to a broader range of sequences with more mismatches.

Of the 33 validated TFBSs known to function as *cis*-regulatory elements in bacterial BGCs (Table 1), 21 (64%) received PWM scores lower than the PWM threshold, representing the minimal score observed among the TFBSs used to construct the PWMs (Fig. 1b). Given that motif-based prediction tools typically apply stringent threshold scores to reduce false positives [3], these findings suggest that more than half of functional TFBSs in BGCs would likely be missed by conventional motif-based approaches. Moreover, among the remaining 12 TFBSs scoring above the PWM thresholds, 7 were associated with a single TF, AdpA, the regulator with the lowest IC (Fig. 1a). While AdpA’s relaxed sequence specificity enables detection of more functional sites, its short binding motif, with only modestly conserved G and C nucleotides (Fig. 1a), poses a major challenge. In high-GC *Streptomyces* and other *Actinomycetota* genomes, such motifs are frequent by chance alone, increasing the number of false positives. Consequently, although functional AdpA binding sites may surpass the PWM threshold, distinguishing them from spurious matches is especially difficult, severely limiting the utility of motif-based prediction for this TF.

### COMMBAT: integration of genomic and functional data into the TFBS scoring method

**Fig. 2.**
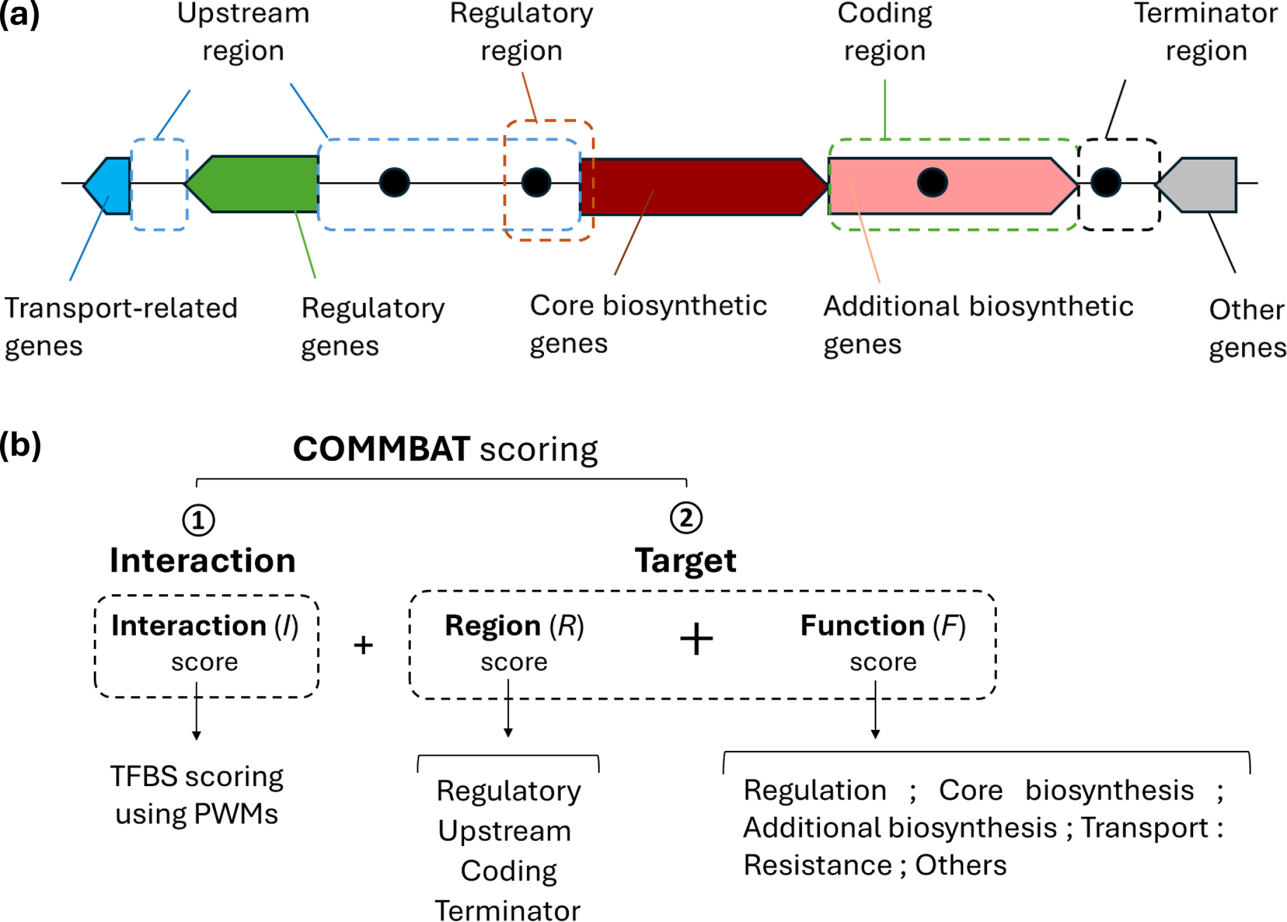
Genomic and functional data integration into the COMMBAT scoring method. (**a**) Definition of the different types of regions and the different genes’ functional categories in BGCs. Upstream region: intergenic region between a start and a start or a stop codon; coding region: between a start and a stop codon; terminator region: intergenic region between two stop codons; regulatory region: region encompassing both the upstream and coding regions of a gene where most TFBS are found. The gene functions are colour-coded according to the scheme applied across MIBiG entries [38]. (**b**) Proposed integration of genomic (region) and functional (function) data into the COMMBAT scoring method.

To enhance the accuracy of functional TFBS prediction within bacterial BGCs, we incorporated both genomic context and functional annotations into the scoring methodology. For a TFBS to be functional – whether for transcriptional activation or repression – it typically has to reside within the ‘regulatory region’ [7], which includes promoters and other expression control elements. In contrast, TFBSs predicted within intergenic regions located between two stop codons (referred to as ‘terminator regions’) are unlikely to play a regulatory role, as such regions generally do not contain promoter elements or influence transcription initiation. Based on the distribution of TFBSs within BGCs [7], we previously defined four distinct genomic regions within gene clusters: the coding region, the upstream region, the regulatory region and the terminator region (Fig. 2a).

A meta-analysis of 328 experimentally validated TFBSs from 91 TFs regulating 75 BGCs revealed that 92% of these sites are located between −371 and +94 nt relative to the translational start codon of a gene [7]. This strongly supports the functional relevance of TFBSs within the defined regulatory region (extending from positions −400 to +100 nt). In contrast, none of the 328 TFBSs were located in terminator regions [7], further reinforcing their limited or negligible regulatory significance. Functional TFBSs in BGCs have also been identified within the coding sequence and upstream of the bounds of the regulatory region, though at a very low occurrence (8%) [7]. This classification provides a biologically grounded framework for prioritizing predicted TFBSs.

Another essential factor to incorporate into the TFBS scoring method is the function of the gene (or the group of genes when organized in operons) potentially regulated by the predicted TFBS. Bacterial BGCs typically contain genes involved in various roles, including biosynthesis, modification, transport and self-resistance to the compound produced. They may also include genes of unknown function and regulatory genes. Among these categories, TFBSs associated with regulatory genes and core biosynthetic genes are considered to have the highest impact on natural product biosynthesis. Indeed, core biosynthetic genes encode enzymes directly involved in constructing the metabolite’s backbone, and regulatory genes encode TFs that often regulate the expression of the entire cluster, ensuring the coordinated production and export of the bioactive metabolite. Consequently, a putative TFBS located in the regulatory region of a cluster-situated TF is particularly significant, as it would imply a ‘one-for-all regulatory strategy’ [7]. Indeed, if functional, such a site is likely to indirectly influence the expression of all genes within the BGC, highlighting the potential regulatory impact of TFBSs positioned upstream of regulatory genes.

Importantly, genes within bacterial BGCs are often organized into operons. Consequently, a TFBS associated with one gene can influence the expression of downstream genes transcribed in the same direction. Identifying operon structures is therefore critical for predicting how regulatory inputs affect multiple genes simultaneously. To assess the functional impact of a TFBS, it is therefore essential to identify the roles of all genes under its control. For instance, there is a major difference between a TFBS located upstream of a single gene with an unknown function and one upstream of a transcriptional unit where only the first gene is uncharacterized, while downstream genes include essential functions such as core biosynthetic genes encoding enzymes that form the backbone of the metabolite. In the latter case, the TFBS is more likely to have meaningful regulatory significance, despite the uncertainty surrounding the first gene.

### COMMBAT score calculation

Based on the transcriptional architecture from our previous meta-analysis on 328 experimentally validated TFBSs from 91 TFs regulating 75 BGCs [7], we developed the COMMBAT scoring methodology to predict the likelihood of a TFBS being involved in the regulation of a BGC (Fig. 2b). The COMMBAT scoring method includes two key components: the *interaction score* and the *target score* and is calculated according to the expression:


$$COMMBAT\;score\;=\frac{1}{2}\left[I+T\right]=\frac{1}{2}[\frac{I_{TFBS}}{I_{max}}+\frac{R+\max\left(F\right)}{2}]\;$$

where

- *I* is the *interaction score*, evaluating the binding affinity between a TF and its predicted binding site. *I* is calculated as the ratio of the PWM score of a predicted TFBS (*I*_TFBS_) to the maximum PWM score (*I*ₘₐₓ). Scores of predicted TFBS are thus normalized on a scale from 0 to 1, where a score of 1 corresponds to the consensus sequence, representing the highest binding affinity for the TF. The PWM sequence scoring method is calculated according to the expression described by Hertz and Stormo [2].
- *T* is the *target score* which incorporates two aspects: (i) The *region score (R)* which reflects the genomic location or type of region bound by the TF, and (ii) the *function score (F)*, which considers the functional classes of genes within BGC that are predicted to be regulated by the TF (Fig. 2b).

The *region score R* of a TFBS is assigned based on its genomic location according to the results of Ribeiro Monteiro *et al*. [7]. In that study, 92% of TFBSs were located between nucleotide positions −371 and +94 relative to the translational start codon. Accordingly, we defined the regulatory region as spanning from positions −400 to +100 nt. A score of 1.0 (maximum) was assigned to TFBSs located in this region, reflecting their strong enrichment and biological relevance. In contrast, sites located further upstream (beyond −400 nt) or deeper within coding sequences (beyond +100 nt) were given a reduced score of 0.3, as only a small fraction of TFBSs were observed in these positions. Finally, terminator regions were assigned a score of 0.0, since no TFBSs were detected there. This scoring system thus directly mirrors the experimentally observed distribution of functional binding sites.

The *function score F* estimates how likely a predicted TFBS is to influence the production levels of the natural product associated with a BGC, based on the function of the gene(s) it potentially regulates. *F* is derived by analysing the gene organization associated with the TFBS to predict co-transcribed genes and their functional categories, which may include regulatory, core biosynthetic, additional biosynthetic, transport, resistance or other. Co-transcribed genes in BGCs are generally located at a distance spanning from −90 to +170 nt relative to the translational stop codon of the upstream gene [7]. The selected *F* score, max(*F*), is based on the gene within the transcription unit that is predicted to most significantly influence the expression or product yield of the BGC. Scores of gene functional categories have been deduced from our previous meta-analysis of over 300 TFBSs [7]. This analysis showed that TFs display a clear hierarchy of preferential targets. Regulatory genes were most frequently targeted, which justified assigning them the maximum value of 1.0. Biosynthetic genes were the next most enriched category: within these, core biosynthetic genes (which encode enzymes responsible for constructing the metabolite backbone) were given a higher value (0.8) than additional biosynthetic genes (0.5), which often act downstream and are frequently co-transcribed in operons. In contrast, transporter genes and self-resistance genes are only rarely targeted and therefore received a lower score (0.2). Finally, other genes, which are seldom targeted and often have unknown functions, were assigned the lowest value (0.1). These values therefore reflect the experimentally observed frequency of TF targeting across functional categories.

After testing several formulae for the COMMBAT scoring, we opted for a balanced approach in which the interaction score contributes 50% of the final score, with the region and function scores contributing 25% each. This formula ensures that the binding affinity remains the dominant factor in the prediction. For the region and function scores, we consider that both are equally important, given that both are necessary for proper BGC expression.

### Comparison of the interaction and the COMMBAT scores

**Fig. 3.**
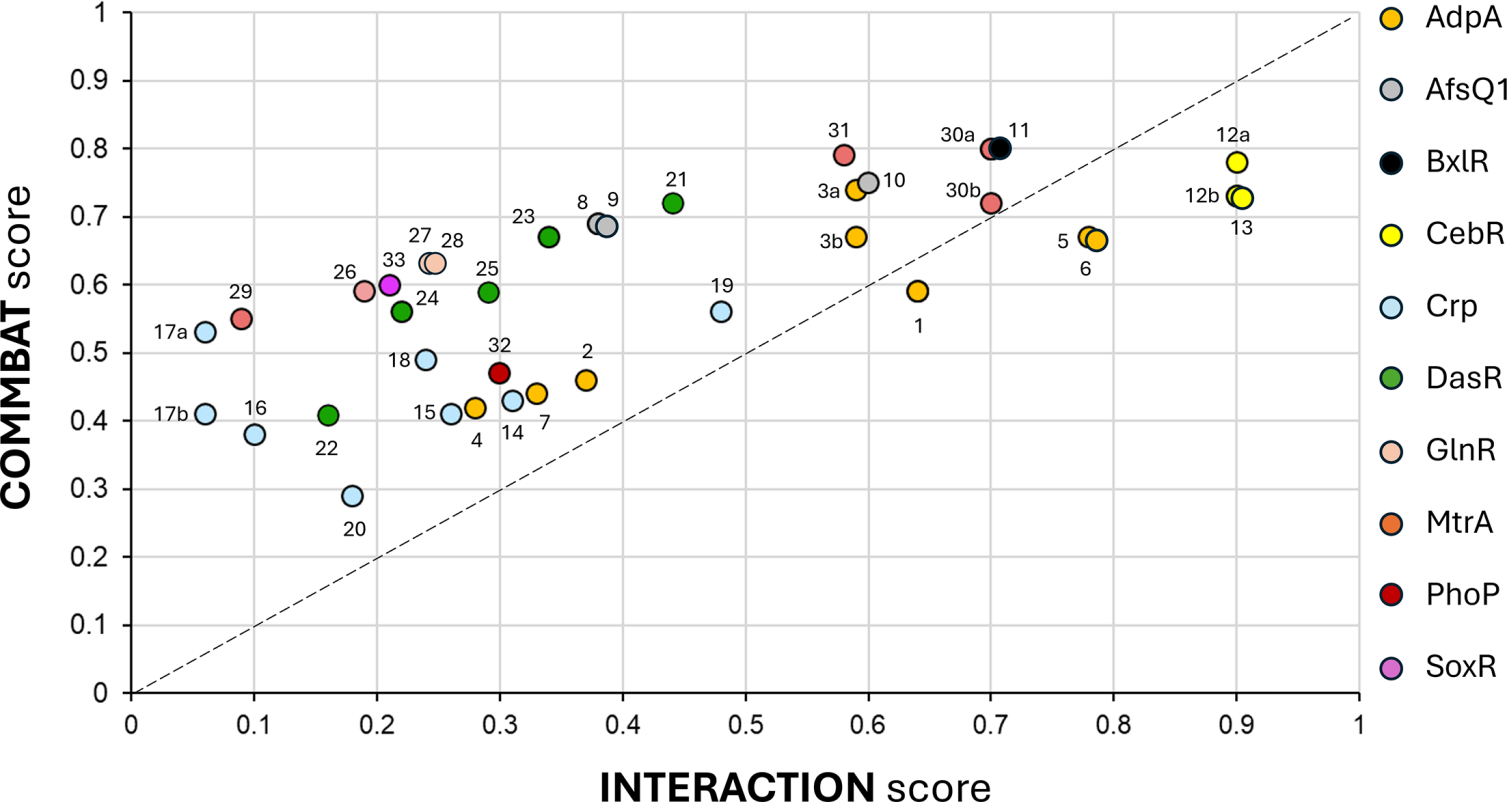
Comparison of the interaction and the COMMBAT scores for each TFBS. Each circle represents a TFBS colour-coded according to their respective TF from Table 1. Note that most TFBSs are positioned above the line of equality (*y*=*x*), indicating higher scores assigned by COMMBAT compared to the interaction method. ‘a’ and ‘b’ refer to two separate COMMBAT scores for TFBSs located in an intergenic region between two divergently transcribed genes.

As shown in Fig. 1, a substantial proportion of TFBSs associated with bacterial BGCs could not have been predicted using motif-based approaches alone. We evaluated whether the COMMBAT score, which integrates both genomic context (region score) and functional relevance (function score), enhances the prediction reliability of these functional TFBSs compared to the interaction score based only on motif similarity scoring using PWM. It has to be noted that in cases when a TFBS is located in an intergenic region between two divergently transcribed genes, COMMBAT generates two separate scores, one for each gene, since the COMMBAT scoring incorporates gene function scores. Fig. 3 shows that the vast majority of TFBSs (83%) have a higher COMMBAT score compared to their corresponding interaction score. On average, the COMMBAT score exhibits a +0.17 score improvement, with the maximum observed increase reaching +0.47 (TFBS #17a). Table 1 highlights the contribution of each component of the target score to the overall score of the predictions. Among the TFBSs analysed, 74% were located within regulatory regions, the only region that contributes positively to the region score (*R*). Additionally, 66% of TFBSs were associated with either the ‘Regulatory’ (40%) or ‘Core Biosynthesis’ (26%) functional categories, both of which provide the strongest positive contributions to the function score (*F*).

We subsequently analysed instances where the COMMBAT scoring method assigned lower scores to functional TFBSs. The most significant reduction, a decrease of 0.17, was observed for a CebR binding site located within the coding region of the core biosynthetic gene *txtB* (TFBS #13 in Fig. 3 and Table 1 [19]), which is essential for thaxtomin A production. Another CebR binding site within the thaxtomin BGC also exhibited a reduced score under the COMMBAT approach. This site is situated upstream of both *txtR* and *txtA*, at positions −786 nt and −900 nt relative to their respective start codons [19], placing it beyond the defined regulatory region boundaries (−400 to +100 nt), thereby negatively impacting the COMMBAT score (TFBSs #12a and #12b in Fig. 3 and Table 1). For CebR, the atypical locations of these TFBSs – either within coding sequences or far upstream of translational start sites – are responsible for the score reductions observed with the COMMBAT method. However, these unconventional TFBS positions are conserved across all *Streptomyces scabiei*-related strains harbouring the thaxtomin BGC [20], underscoring their functional significance despite their non-canonical locations potentially involving DNA looping. Other two instances of score reduction involve the TFBSs of AdpA (#5 and #6 in Fig. 3 and Table 1). These reductions are attributed to the TFBSs being located in the regulatory region of a gene annotated as having an ‘other or unknown’ function. However, this gene (SGR_5931) actually encodes StrR, the regulator of the streptomycin biosynthesis operon [21], and should be classified under the ‘Regulatory’ functional category. The reduced COMMBAT scores for these TFBSs are therefore the result of a misclassification by the MIBiG database. When correctly classified, their COMMBAT scores should be 0.89 and 0.82, which exceed their respective interaction scores of 0.78 and 0.64.

**Fig. 4.**
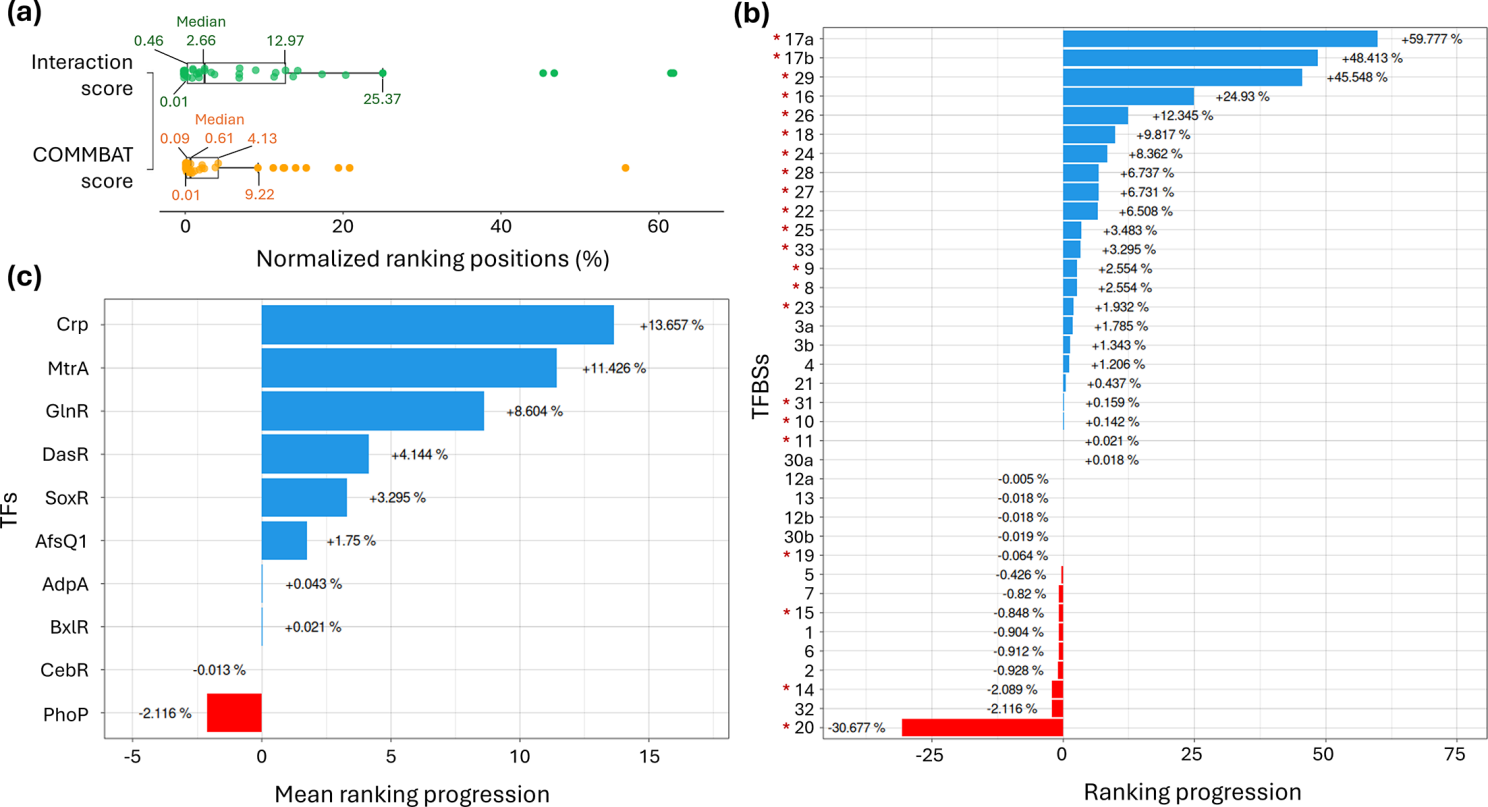
Effect of the COMMBAT scoring methodology on the ranking progression of functional TFBSs within BGCs. (**a**) Boxplots showing the distribution of normalized rankings (in %) for experimentally validated TFBSs according to the methodology used (interaction and COMMBAT scoring). The edges in the boxplot indicate the first and third quartiles, and the median is the centre line. (**b**) Ranking progression (in %) for the COMMBAT scoring for each functional TFBS from Table 1. Positive and negative progressions are coloured in blue and red, respectively. TFBSs that had an interaction score below the PWM detection threshold (Fig. 1b) are marked with an asterisk. (**c**) Mean ranking progression (in %) for the COMMBAT scoring for each studied TF. Positive and negative progressions are coloured in blue and red, respectively.

We next evaluated the ability of the COMMBAT scoring method to distinguish functional TFBSs from a large background of false positives. The underlying idea is that if our proposed scoring method is more reliable, it should consistently prioritize experimentally validated TFBSs, ranking them higher relative to random matches. To test this, we selected ten TFs with experimentally validated binding sites (Table 1). Using their PWMs, we scanned all 802 *Streptomyces* BGCs from the MIBiG database (v. 4.0) [15], along with non-*Streptomyces* BGCs regulated by the same TFs. For each TF, all sequence matches of the same length as its TFBS and with a PWM score above zero were retained, generating a large sequence pool enriched in false positives. Within these pools, the validated TFBSs were ranked according to either the classical interaction score or the COMMBAT score. To allow direct comparison between methods, ranks were normalized to the total number of predicted sites and expressed as percentages (Table 1, Fig. S1). Overall, the functional TFBSs analysed in this study were ranked higher (indicating greater reliability) when using the COMMBAT scoring method compared to the interaction scoring method, as reflected by higher median ranking position values of 0.61 and 2.66%, respectively (Fig. 4a). Moreover, the interquartile range (representing 50% of TFBS) and the overall distribution across all four quartiles covered a significantly narrower portion of the ranking spectrum with the COMMBAT method (from 0.09 to 4.13%) compared to the interaction score method (from 0.46 to 12.97%) (Fig. 4a). This concentration of functional TFBSs among top-ranked positions suggests a higher signal-to-noise ratio and therefore improved prediction reliability.

The ranking progression of each functional TFBS between the COMMBAT and interaction scoring methods is provided in Table 1 and illustrated in Figs 4(b) and S1. Among the 33 TFBSs analysed, 23 (70%) showed a positive ranking progression using the COMMBAT scoring method compared to the interaction scores calculated using only sequence similarity (Table 1). Importantly, 17 out of the 21 TFBSs with interaction scores below the PWM detection threshold (Fig. 1b) showed a positive ranking progression (Fig. 4b, TFBSs with asterisk, Table 1). The most significant progression was observed for TFBS #17a, which also showed the highest score improvement (Fig. 3). For TFBSs that show decreased ranking positions, the decreases are relatively limited (<2.2%), except for TFBS #20. The latter shows a higher COMMBAT score than the interaction score (Fig. 3, Table 1) but still presents an important ranking decrease of 30.7%. This could be explained by an overall low COMMBAT scoring (0.29) with low region and function scores (0.3 and 0.5, respectively), which negatively impacts the TFBS ranking compared to other sequences that have higher region and function scores. The mean ranking progression for each TF was also calculated and illustrated in Fig. 4(c). In total, eight out of ten TFs show an overall positive ranking progression with the COMMBAT scoring. The best progression was observed for Crp (13.7%). Once again, the decreases for the mean ranking progression are relatively limited, with CebR having a mean decrease of 0.013% and PhoP the highest decrease of 2.1%.

### Limitations and future development

The developed COMMBAT scoring methodology provides an accurate and functionally meaningful framework for identifying regulatory elements within BGCs. However, the proposed methodology also presents several limitations. First, although the COMMBAT scoring methodology enhances the reliability of poorly conserved TFBSs within BGCs, we acknowledge that binding affinity remains the most critical factor for TFBS prediction. Therefore, we developed a website interface (www.commbat.uliege.be; website manuscript in preparation) to allow the scientific community to use this methodology and to have access to the individual interaction and target scores in addition to the composite COMMBAT score. Access to these individual scores allows expert users to adjust prioritization based on prior knowledge of TF specificity. For example, if evidence indicates that a TF tolerates only one or two mismatches to its consensus motif, the user can choose to rank candidates by interaction score alone or evaluate its contribution within the overall COMMBAT score.

Second, the COMMBAT scoring formula is based on the current state of knowledge of the transcriptional architecture of bacterial BGCs. Most TFBSs within BGCs have been identified using electrophoretic mobility shift assays (EMSA) and footprinting assays, which primarily focus on upstream regions of genes, introducing a bias towards this region. In the future, non-targeted genome-wide approaches such as DAP-seq or ChIP-seq are expected to expand the identification of TFBSs, including those located within coding sequences. Consequently, the scoring will likely evolve as more studies elucidate the mechanisms involved in BGC regulation.

Third, the COMMBAT methodology is based on the PWM scoring to calculate the interaction score. However, the PWM approach is not appropriate when handling binding site motifs that have spacer regions. Thus, the use of COMMBAT is currently limited to the predictions of TFBSs that have ungapped binding sites. In future developments, we plan to integrate profile hidden Markov models to make predictions of TFBSs which have spacer regions [7].

Finally, the methodology considers each TFBS separately when calculating the COMMBAT score. If there are multiple TFBSs regulating a gene or a cluster, each TFBS will have its own separate COMMBAT score. In future developments, we may explore aggregating scores from multiple binding sites within a single BGC. However, this alternative scoring approach may not necessarily improve the predictions, as many global TFs primarily regulate the pathway-specific regulator of the BGC via a single TFBS, which in turn controls expression of all cluster genes, rather than acting on multiple TFBSs across all transcription units. We are currently evaluating this approach to ensure that BGCs containing only a single but functional TFBS are not unduly penalized.

## Conclusions

In this study, we addressed a key limitation of conventional motif-based approaches for TFBS prediction within the context of bacterial BGCs. Our findings demonstrate that a significant proportion of functional TFBSs within BGCs are poorly conserved and fall below the detection threshold of standard PWM-based methods. This limitation hampers efforts to decode the regulatory architecture underlying BGC expression control, essential knowledge to unveil the environmental signals linked to natural product biosynthesis, and to unlock the vast reservoir of uncharacterized cryptic natural products. To overcome this challenge, we developed the COMMBAT scoring methodology that integrates both binding affinity (via interaction score) and biological context (via genomic region and gene function). Our results show that the regulatory region positioning associated with genes encoding key functions of BGC activation significantly enhances the prediction reliability of functional TFBSs, with over 83% of sites analysed receiving higher composite scores than when evaluated by motif similarity alone. Furthermore, when comparing the ranking of these TFBSs for both methods in a random set of sequences, we observed that 70% of sites have a better ranking with the COMMBAT methodology. By incorporating biologically grounded contextual information, COMMBAT provides a more accurate and functionally meaningful framework for identifying regulatory elements within specialized metabolic pathways. However, the proposed method strongly depends on the annotation quality of the genes’ functions, and thus wrong annotations can lead to reduced COMMBAT scores (see TFBSs #5 and #6). To facilitate its use by the broader scientific community dedicated to natural product discovery, a web interface is provided (https://commbat.uliege.be; website manuscript in preparation), enabling users to predict TFs – and their associated environmental signals – that may regulate BGC expression based on COMMBAT scores. In conclusion, this refined prediction capability is expected to accelerate the discovery and rational activation of silent or poorly expressed BGCs, offering new avenues in natural product discovery.

## Supplementary material

10.1099/mgen.0.001512 Uncited Supplementary Material 1.

## Abbreviations

BGC: biosynthetic gene cluster
COMMBAT: COnditions for Microbial Metabolite Activated Transcription
EMSA: electrophoretic mobility shift assay
IC: information content
PWM: position weight matrix
TF: transcription factor
TFBS: transcription factor binding site
