# Supplementary material for: Enhanced prediction of expression control in bacterial biosynthetic gene clusters via genomic and functional data integration
Source: Microb Genom. 2025 Oct 15;11(10):001512. doi: 10.1099/mgen.0.001512 (PMC12527672; doi:10.1099/mgen.0.001512)
Supplement: Uncited Supplementary Material 1. [file mgen-11-01512-s001.pdf]

Supporting information of

COMMBAT: enhanced prediction of expression control in biosynthetic gene clusters via genomic and functional data integration

Silvia Ribeiro Monteiro[1], Sébastien Rigali[1,*].

[1]InBioS – Center for Protein Engineering, University of Liège, Institut de Chimie, Liège B-4000, Belgium

[*]Corresponding author: srigali@uliege.be

Table of Contents

Supplementary Figure 1. 2

Position Weight Matrices. 7

Supplementary Figure

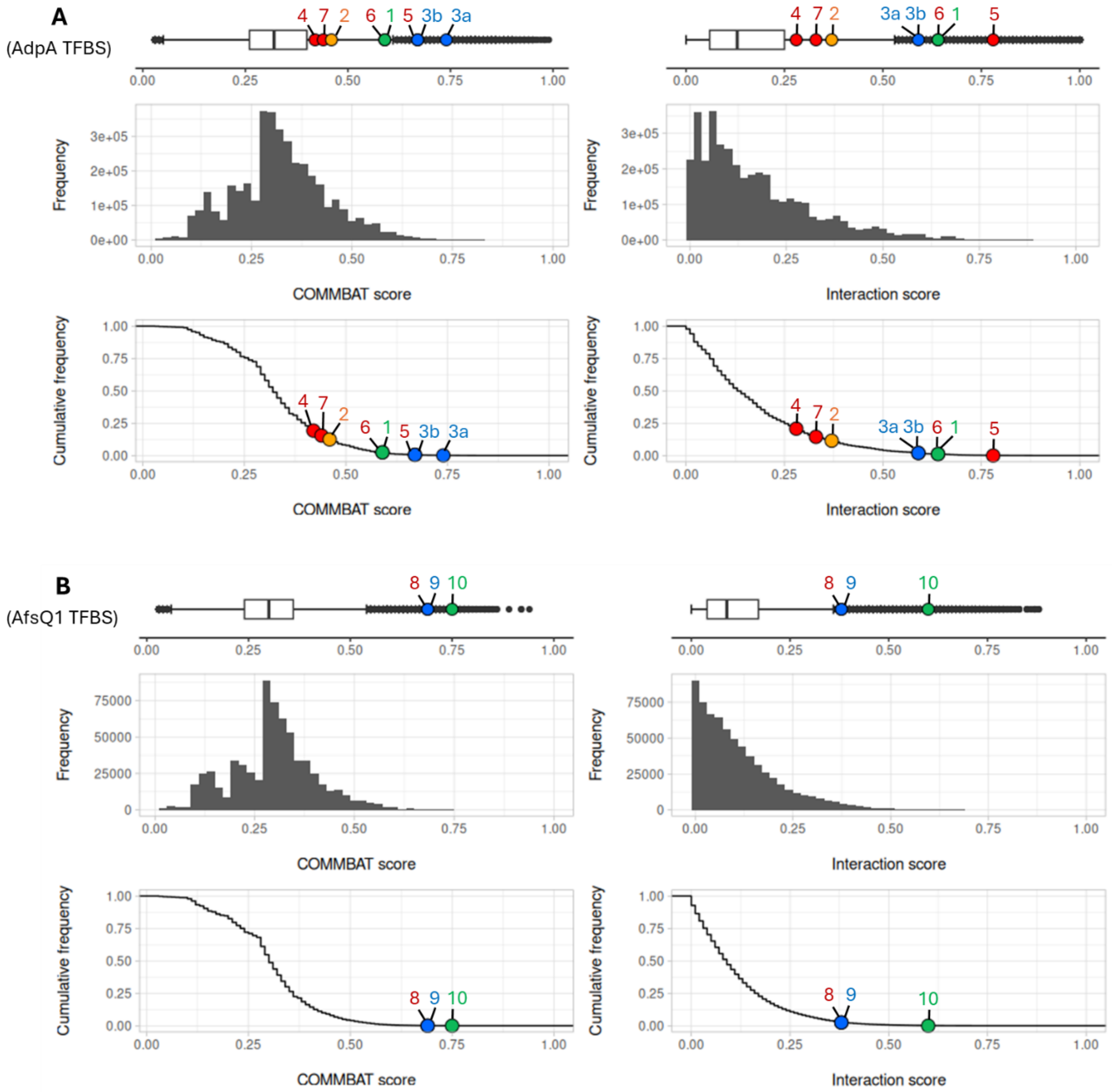

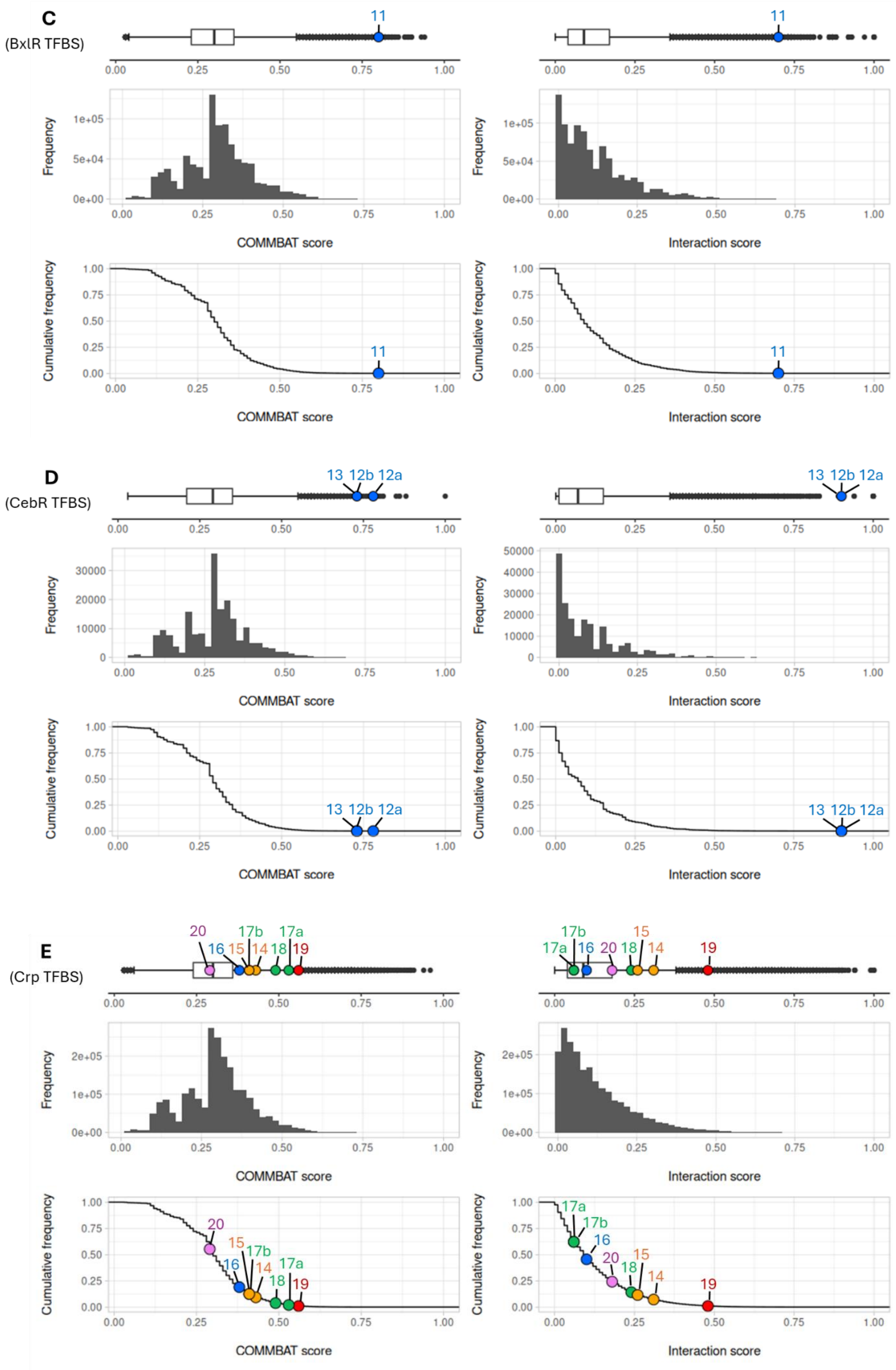
C
(BxlR TFBS)
11
Frequency
1e+05
5e+04
0e+00
COMMBAT score
Interaction score
Cumulative frequency
0.00
0.25
0.50
0.75
1.00
D
(CebR TFBS)
13 12b 12a
30000
20000
10000
50000
40000
E
(Crp TFBS)
20
16 15 14 18 19
17b
17a
2e+05
1e+05

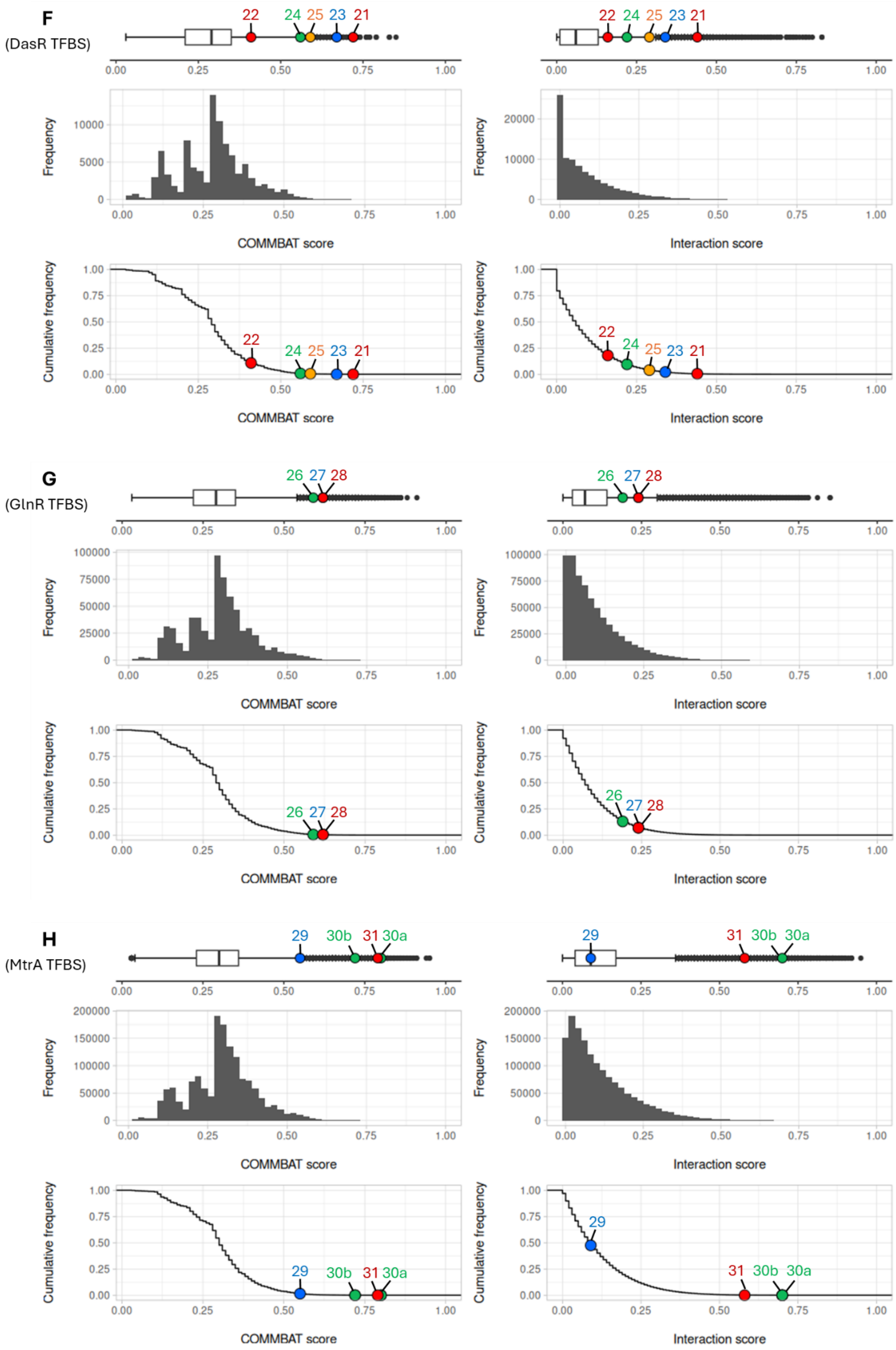
F
(DasR TFBS)
22
24 25 23 21
22 24 25 23 21
0.00 0.25 0.50 0.75 1.00
Frequency
10000
5000
0
20000
10000
0
COMMBAT score
Interaction score
Cumulative frequency
1.00 0.75 0.50 0.25 0.00
COMMBAT score
Interaction score
G
(GlnR TFBS)
26 27 28
Frequency
100000
75000
50000
25000
0
COMMBAT score
Interaction score
Cumulative frequency
COMMBAT score
Interaction score
H
(MtrA TFBS)
29 30b 31 30a
29 31 30b 30a
Frequency
200000
150000
100000
50000
0
COMMBAT score
Interaction score
Cumulative frequency
COMMBAT score
Interaction score

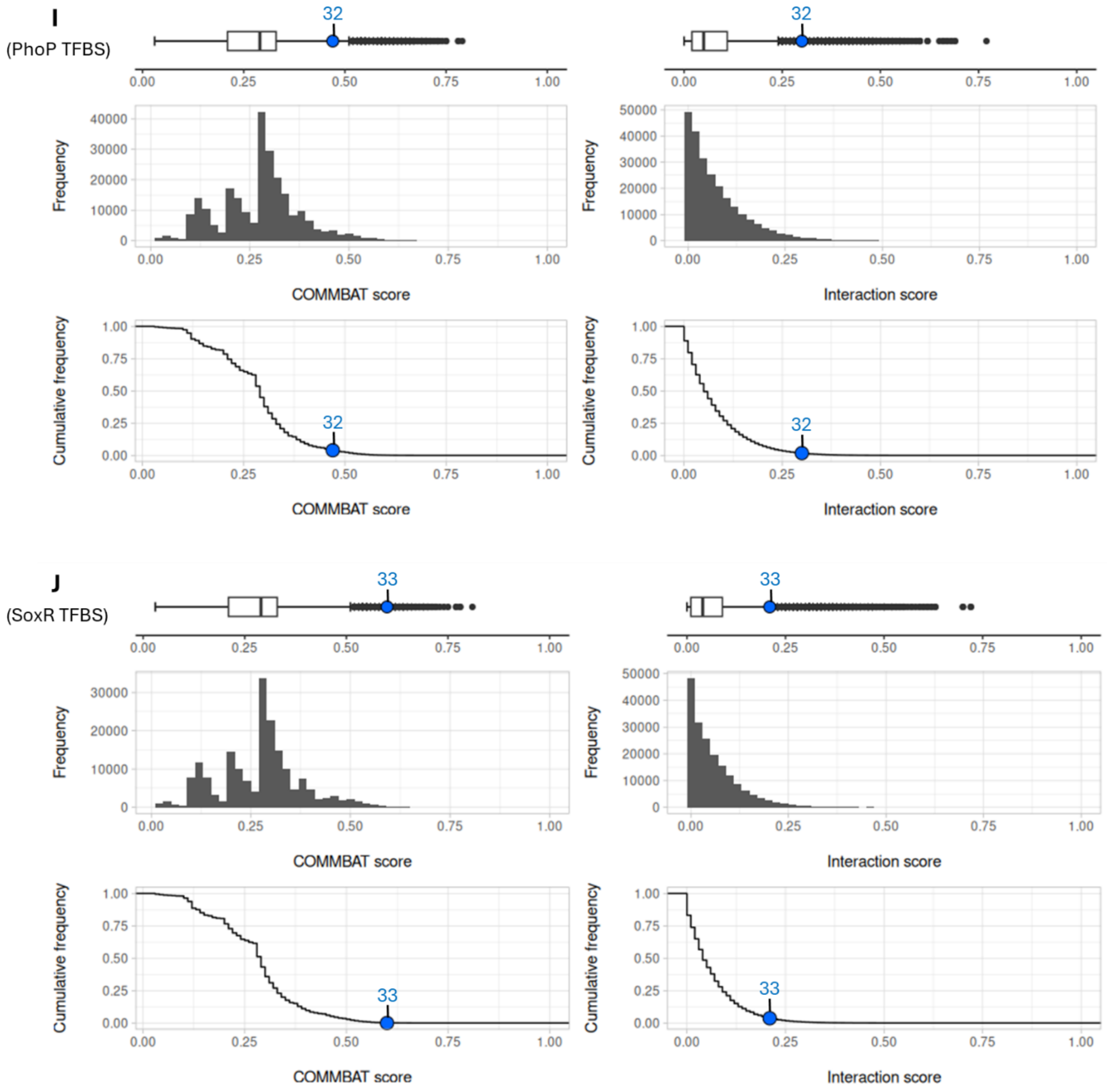

Supplementary Figure 1. Distribution of COMMBAT (left side) and Interaction scores (right side) for the set of sequences that match known TFBSs with a similarity PWM score above 0 for the 10 studied TFs: AdpA (A), AfsQ1 (B), BxlR (C), CebR (D), Crp (E), DasR (F), GlnR (G), MtrA (H), PhoP (I) and SoxR (J). Known TFBSs are represented with colored dots (colors representing different BGCs) and indexed according to Table 1. The distribution is illustrated with different visualizations: boxplot (upper panel), frequency of sequences (middle panel), and cumulative frequency of sequences (lower panel).

Position Weight Matrices. All sequences used to generate the 10 PWM used in this study are listed below:

1. **AdpA**

>SGR_4151_[S.griseus]

TGGCGGGTTC

>SGR_2393_[S.griseus]

CGGCCGGAAT

>SGR_2393_2_[S.griseus]

GGGCCGAATA

>SGR_3655_[S.griseus]

TGGCGCGATC

>SGR_3655_2_[S.griseus]

CGTCGGCAAG

>SGR_3655_3_[S.griseus]

TGGCCATTTC

>SGR_3655_4_[S.griseus]

AGCGGCGTTA

>SGR_3655_5_[S.griseus]

TGGCCAGATT

>SGR_3655_6_[S.griseus]

GGACCGGTCT

2. **AfsQ1**

>SCO0674_[S.coelicolor]

GTCACCGCCGGGTCAC

>SCO0812_[S.coelicolor]

GTCACGACCGAGTCAC

>SCO1619_[S.coelicolor]

GTCACATCGATGTCAC

>SCO1839_[S.coelicolor]

GAGACAAGGTTGTGAC

>SCO1947_[S.coelicolor]

GTCACGAACCGATCTC

>SCO2527_[S.coelicolor]

GAGACCTTTCCGTGAC

3. **BxlR**

>bxlE_[S.thermoviolaceus]

CGAAACATTCG

>stxI_[S.thermoviolaceus]

CGAAACTTTCG

>stxII_[S.thermoviolaceus]

CGAACCTTTCG

>xlnC_[Streptomyces_sp._ECS]

CGAAAGTGTTG

4. **CebR**

>SGR199_secreted_cellulose-binding_protein_[S.griseus]

TGGGAGCGCTCCCA

>SGR199_secreted_cellulose-binding_protein_[S.griseus]

CGGGAGCGCTCCCC

>SGR217_secreted_pectate_lyase_[S.griseus]

TGGGAGCGCTCCCG

>SGR1971_secreted_protein_of_unknown_function_[S.griseus]

TGGGAGCGCTCCCC

>SGR3391_acetyltransferase_[S.griseus]

CGGGAGCGCTCCCG

>bglC_SGR199_[S.griseus]

AGGGAGCGCTCCCA

>SGR6927_hypothetical_protein_[S.griseus]

TTGGAGCGCTCCCA

>SGR6928_secreted_cellulase_[S.griseus]

TGGGAGCGCTCCAA

>bglC_SCAB_RS24385(51081)_[S.scabiei]

TGGAAGCGCTCCCA

5. **Crp**

>crp_SCO3571_[S.coelicolor]

GTGACAGATCACAC

>SCO4561-4562_[S.coelicolor]

GTGGCGCAGGTCAC

>SCO2976-2977_[S.coelicolor]

GTGATGCGGCCGAC

6. **DasR**

>crr_SCO1390_[S.coelicolor]

TGTGGTCTAGACCTCT

>malX2_SCO2905_[S.coelicolor]

ACTGGTGTAGACCAGT

>nagE2_SCO2907_[S.coelicolor]

ACCGGTCTACACCACT

>nagE2_SCO2907_[S.coelicolor]

AGTGGTGTAGACCACC

>ptsH_SCO5841_[S.coelicolor]

ACTGGTCTAGACAACT

>ptsH_SCO5841_[S.coelicolor]

ACTGGTCTAGACAAGA

>dasR_SCO5231_[S.coelicolor]

ACTGGTCTACACCATT

>nagB_SCO5236_[S.coelicolor]

TGTGGTTTAGACCAAT

>ptsH_SCO5841_[S.coelicolor]

AGTTGTCTAGACCAGT

>ptsH_SCO5841_[S.coelicolor]

TCTTGTCTAGACCAGT

>chiD_SCO1429_[S.coelicolor]

ACTGGTCTAGTCCTCC

>chiD_SCO1429_[S.coelicolor]

AATGGTCCGAACCATT

>chiI_SCO1444_[S.coelicolor]

ACTGGTCTAGTCCTCT

>chiI_SCO1444_[S.coelicolor]

ATTGGTCCATACCTAT

>chiH_SCO6012_[S.coelicolor]

AATGGTCTGGACCAGA

>chiH_SCO6012_[S.coelicolor]

GGTGGACTGGACCACC

>chiH_SCO6012_[S.coelicolor]

ATGGGACTAGACCAAT

>SCO6345_chitin-binding_protein_[S.coelicolor]

TAAGGTCTAGACCTGC

>SCO6345_chitin-binding_protein_[S.coelicolor]

GTAGGTCTAGACCTGC

>chi_SCO7225_[S.coelicolor]

TCAGGTCTAGACCTGT

>chi_SCO7225_[S.coelicolor]

TATGGTCTAGACCTGA

>chi_SCO7225_[S.coelicolor]

CCTTGTCTAGACCAAT

>chiE_SCO5954_[S.coelicolor]

ATTGGTCCAGACCTTC

>chiE_SCO5954_[S.coelicolor]

ACAGGCGCAGACCACC

>chiA_SCO5003_[S.coelicolor]

GGTGGTCCAGACCAAT

>chb3_SCO0481_[S.coelicolor]

TATGGTCTAGTCCAAC

>chb_SCO2833_[S.coelicolor]

GCAGGTCTAGACCAAG

>SC02943_beta_N-acetylglucosaminidase_[S.coelicolor]

AGAGGTCTGAACCAAT

>chiJ_SCO2503_[S.coelicolor]

AAAGGTCTGGACCACA

>chiJ_SCO2503_[S.coelicolor]

CTTGGTCCAGACCTCT

>chiJ_SCO2503_[S.coelicolor]

TCTGGACCACAGCACT

>chb_SCO2833_[S.coelicolor]

ACATGTCCATACCAAA

>nagE1_SCO2906_[S.coelicolor]

ACTGGTCTACACCAGT

>nagE1_SCO2906_[S.coelicolor]

ATCGGTCTGCACCAGT

>nagE1_SCO2906_[S.coelicolor]

CAAGGTGTAGACCTCT

>nagE2_SCO2907_[S.coelicolor]

ACAGGTCTACACCACT

>SCO5230_putative_integral_membrane_protein_[S.coelicolor]

TCTGGTCTAGTCCTGG

>chiC_SCO5376_[S.coelicolor]

ATAGGTCTGGACCAAT

>chiC_SCO5376_[S.coelicolor]

AAAGGTCTGGACCATA

>chiB_SCO5673_[S.coelicolor]

ATTGGTCTGGACCAAA

>SCO6032_putative_hydrolase_[S.coelicolor]

CTTGGTCTAGTCCATT

>SCO6300_putative_secreted_hydrolase_[S.coelicolor]

ATAGGTCTAGACAAAA

>SCO6300_putative_secreted_hydrolase_[S.coelicolor]

AGAGGTCTAGACAAAA

>glyS_SCO2504-05_[S.coelicolor]

AGTGGTCTGCACCTGG

>SCO3679_hypothetical_protein_[S.coelicolor]

TGTTGTCTAGTCCAAT

>SCO3490_transposase_[S.coelicolor]

AATCGTCAAGACCTGT

>SCO4699_putative_Rhs_protein_[S.coelicolor]

AGAGGTCGACACCCGT

>pglX_SCO6627_[S.coelicolor]

CACGGTGTAGACATCA

>SCO3262_hypothetical_protein_[S.coelicolor]

ACAGGAGGACACCATT

>WHTH_GntR_SCO7056_[S.coelicolor]

ATTGGTCTAAACCAGC

>SCO5239_histidine_kinase_[S.coelicolor]

AGTGGTCTAGTCCACA

>SCO5550_putative_transcriptional_regulator_[S.coelicolor]

ACTGGTCTAAACCACA

>nagKA_SCO4284-85_[S.coelicolor]

AGAGGTCTAGTCCACT

>nagKA_SCO4284_[S.coelicolor]

GGTGGTGTAGACCTTA

>dasA_SCO5232_[S.coelicolor]

CTTGGTCTAGTCCATA

>ngcE_SCO6005_[S.coelicolor]

AGTGGACTATACCTGT

>nagE2_SACE_2058_[S.erythraea]

TCCGGTCTAAACCAGT

>nagKA_SACE_7183-84_[S.erythraea]

GTTGGTGTAGACCACT

>pstI_SACE_7368_[S.erythraea]

CATGGTCTAGACCATA

>nagB-II_SACE_0498_[S.erythraea]

CCAGGTCTAGACCAAT

>chiD_SACE_6558_[S.erythraea]

TAAGGTCTAGACCACC

>glmS_SACE_6768_[S.erythraea]

ATTGGTCCATACCTCC

>chb_SACE_0110_[S.erythraea]

TTTGGTCCAGACCATG

>chb_SACE_0110_[S.erythraea]

TTTGGTCCAGTCCAAC

>chiC_SACE_3887_[S.erythraea]

ACTGGTCTGGACCAAA

>chiA_SACE_5394_[S.erythraea]

AGTGGTCTAGTCCATT

>hexA_SACE_3851_[S.erythraea]

TTTGGTCTATACCTTT

>chiB_SACE_2232_[S.erythraea]

AGTGGACTGGACCACC

>chiB_SACE_2232_[S.erythraea]

AATGGTCCGGTCCAAT

>chiH_SACE_1076_[S.erythraea]

ATTGGTTTGTACCACC

7. **GlnR**

>glnA_SCO2198_[S.coelicolor]

TTAACTTCTGCGAAAC

>amtB_SCO5583_[S.coelicolor]

GTCACGGCGGCGAAAC

>glnII_SCO2210_[S.coelicolor]

GTAACACGGGGTTCAC

>gdhA_SCO4683_[S.coelicolor]

TTTACATGCGCGTAAC

>nirB_SCO2486_[S.coelicolor]

GTTCCCCGTGGGAAAC

>ureA_SCO1236_[S.coelicolor]

GTAATCAATCGGTCAA

8. **MtrA**

>amtB-a1b1_SCO5583_[S.coelicolor]

GTCACGGCGGCGAAAC

>amtB-a2b2_SCO5583_[S.coelicolor]

GGGCTTCCACCGAAAC

>glnII_SCO2210_[S.coelicolor]

GCAACGGTCGGGAAAT

>nirB_SCO2486_[S.coelicolor]

GATGCGGCCGGGTAAC

>ureA_SCO1236_[S.coelicolor]

GTGACGTGGCCGTCAC

9. PhoP

>pstS_SAV4072_[S.avermitilis]

GTTCACCTTCCGTTCACTCTCG

>phoU-phoRP_SAV3974_[S.avermitilis]

CGTCGCCCAGAGTTCACCTGGG

>pstS_SCO4142_[S.coelicolor]

GTTCACCCGGCGTTCATTTACG

>PhoU-PhoR_SCO4229-SC4228_[S.coelicolor]

CGTCGCCCAGAGTTCACCTTGG

>PhoU-PhoR_2_SCO4229-SC4228_[S.coelicolor]

AGCCAGGGACGGTTCATTTGGC

>pitH2_1_SCO1845_[S.coelicolor]

GTTCACCGTGGCGCCCTGCCCA

>pitH2_2_SCO1845_[S.coelicolor]

GTTCATCTTCCGTTCACCCTGG

>pstCAB-phoS2_SACE_7099_[S.erythraea]

GTTCACCTGGGGTTCACCTCCA

>phoU_SACE_7091_[S.erythraea]

GTTCACCCGCGGTTCACCACGG

>phoRP_SACE_6966_[S.erythraea]

CTTCACCCCCATTTCACCCTGG

10. SoxR

>SAV_5665_[S.avermitilis]

CCTCAAGATTGGTTGAGG

>SAV_3955-56-57_[S.avermitilis]

CCTCAAGCAAGCTTGAGG

>SAV_7218_[S.avermitilis]

CCTCAAGATTGCTTGAGG
